# Protein requirement and its intake in subjects with diabetic foot ulcers at a tertiary care hospital

**DOI:** 10.12669/pjms.344.15399

**Published:** 2018

**Authors:** Nida Sajid, Zahid Miyan, Syed Itaat Hussain Zaidi, Syeda Sara Abbas Jaffri, Mariam AbdeAli

**Affiliations:** 1 Dr. Nida Sajid, FCPS. Fellow Endocrine, Baqai Institute of Diabetology and Endocrinology, Baqai Medical University, Karachi, Pakistan; 2 Dr. Zahid Miyan, MCPS, MD. Assistant Professor of Medicine, Department of Medicine, Baqai Institute of Diabetology and Endocrinology, Baqai Medical University, Karachi, Pakistan; 3 Syed Itaat Hussain Zaidi, FCPS. Associate Professor of Orthopedic Surgery, Dow University of Health Sciences, Karachi, Pakistan. Honorary Orthopedic Surgeon (Visiting), Baqai Institute of Diabetology and Endocrinology, Baqai Medical University, Karachi, Pakistan; 4 Syeda Sara Abbas Jaffri, MSc. Clinical Dietitian, Diet and Education Department, Baqai Institute of Diabetology and Endocrinology, Baqai Medical University, Karachi, Pakistan; 5 Mariam Abde Ali, BS. Clinical Dietitian, Diet and Education Department, Baqai Institute of Diabetology and Endocrinology, Baqai Medical University, Karachi, Pakistan

**Keywords:** Diabetic foot ulcers, Diet, Protein, Requirements, Intake, Pakistan

## Abstract

**Objective:**

To assess the protein intake and requirement among subject with type 2 diabetes having foot ulcers.

**Methods:**

This study was conducted at Baqai Institute of Diabetology & Endocrinology (BIDE), a tertiary care diabetes centre of Karachi, Pakistan among people with type 2 diabetic foot ulcer attending foot clinic from January 2012 to March 2015. The baseline characteristics, dietary intake and laboratory investigations of the study participants were obtained through electronic hospital database “Health Management System” (HMS) based on the 24 hours dietary recall interview. Total grams of protein were calculated from each food group consumed by the subject. Protein intake of the subjects was recorded in mean grams and the protein requirement was calculated according to their body weight. The comparison of intake and requirement of protein choices was done through comparing the mean of both variables. SPSS version 13 was used for analysing the results.

**Results:**

A total of 542 subjects were included in the study, 365 (67.2%) were males and 178 (32.8%) were females. Mena age of the subject was 54.61±10.51 (yrs) with the duration of diabetes and mean body mass index were 14.22±7.98 (yrs) and 26.65±5.38 (kg/m^2^), respectively. The dietary records showed the protein intake of subjects with diabetic foot ulcer is not appropriate when compared to daily requirement. Mean grams of protein intake is 76.87gms in males and 56.84gms in females. On the other-hand protein requirement is much higher than the intake, which is 219.5gms in males and 130.2gms in females.

**Conclusion:**

Dietary counselling should be a part of the treatment among subjects with diabetic foot ulcer to identify their nutritional needs and suggesting them better option to fulfil their protein requirement essential for wound healing process.

## INTRODUCTION

Diabetes mellitus is a group of metabolic diseases associated with long term effect on many organs which results in micro and macro vascular complications.^1^ Diabetic foot ulcer is one of the most common complications of diabetes which often difficult to heal.^2^ Wound healing process consist of a series of interactions between different cell types, cytokine mediator and the extracellular matrix. This process may be successful by the adequate blood supply and nutrients to the site of damaged tissue.^3^,^4^

Nutrition therapy plays a vital role in the management of diabetes and complications related to it.^5^ Poor nutrition before or during the healing process may delay wound healing and impair wound strength.^3^,^6^,^7^ Protein is one of the most important macronutrient as it is essential for wound healing.^8^,^9^ It is required for all stages in healing process including fibroblast proliferation, collagen synthesis, angiogenesis and immune function.^10^,^11^

European Pressure Ulcer Advisory Panel and Agency for Health Care Policy and Research have both recommended to increase intake of protein for wound healing process.^12^,^13^ Inadequate protein intake may result in delay in wound healing process.^14^ Although the effect of inadequate protein intake has been observed in non-diabetic people with wounds, there is very scarce data regarding the frequency of protein deficiency in people with diabetic foot ulcer.^10^

Therefore, the aim of this study was to assess the protein intake in subjects with type 2 diabete having foot ulcers.

## METHODS

This observational study was conducted at Baqai Institute of Diabetology & Endocrinology (BIDE), a tertiary care diabetes centre of Baqai Medical University, Karachi _ Pakistan. To assess the dietary records of the subject with type 2 diabetes having foot ulcer attending the foot clinic department of BIDE from January 2012 to March 2015. Pregnant women, subjects with acute illness, with type 1 diabetes and other complications like nephropathy, liver diseases were excluded from the study. Ethical approval was obtained from the Intuitional Review Board (IRB) of BIDE.

The baseline characteristics, dietary intake and laboratory investigations of the study participants were obtained through electronic hospital database “Health Management System” (HMS) based on the 24 hours dietary recall face to face interview by trained dietician. HMS was designed to record their dietary choices in which all the food intake was obtained from breakfast to bedtime. The number of servings consumed from different food groups like bread & cereal group, fruit group, vegetable group, meat & poultry group and milk group were recorded. The injury factor was assessed using the University of Texas (UT) classification then the terms minor, mild, moderate and severe was given to each accordingly. The requirement was calculated accordingly, 1gm per body weight was decided for minor injury, 1.1 for mild, 1.2 for moderate and 1.5 for severe injury.^15^

### Statistical Analysis

Statistical Package for Social Sciences (SPSS) version 20 was used to analyze the data. Independent t test and chi-square test was applied, to test for statistically significant differences. P-value equal to or less than 0.05 was considered as significant.

## RESULTS

A total of 542 subjects were included in the study, 365 (67.2%) were males and 178 (32.8%) were females. Mean age of the subject was 54.61±10.51 (yrs) with the duration of diabetes 14.22±7.98 (yrs). Mean weight and body mass index of the subject was 72.02±15.58 (kg) and 26.65±5.38 (kg/m^2^), respectively. The mean serum creatinine was 1.13±0.29 (mg/dl) with the mean HbA1c was 10.1±2.4 (%). Mean serum cholesterol, triglyceride High Density Lipoprotein (HDL) and low density lipoprotein were 132.18±33.85 (mg/dl), 116.87±63.83 (mg/dl), 28.95±10.63 (mg/dl) and 77.18±26.87 (mg/dl), respectively (Table-I).

**Table-I:** Baseline characteristics of the study subjects.

Variables	Male	Female	P-value	Overall
N	364	178	-	542
Age (years)	55.32±10.14	53.15±11.12	0.024	54.61±10.51
Height (cm)	168.21±6.92	156.42±6.91	<0.0001	164.34±8.86
Weight (kg)	73.98±14.92	68.02±16.17	<0.0001	72.02±15.58
BMI (kg/m2)	26.08±4.72	27.81±6.39	<0.0001	26.65±5.38
Duration of diabetes (years)	14.45±8	13.75±7.95	0.336	14.22±7.98
Systolic blood pressure (mmHg)	132.4±18.51	137.51±17.11	0.002	134.06±18.21
Diastolic blood pressure (mmHg)	83.38±9.69	80.46±7.53	0.001	82.44±9.14
Serum creatinine (mg/dl)	1.18±0.25	1.02±0.33	<0.0001	1.13±0.29
Serum cholesterol (mg/dl)	129.89±34.06	137.91±32.82	0.069	132.18±33.85
Serum triglyceride (mg/dl)	110.5±59.72	132.89±71.05	0.007	116.87±63.83
HDL cholesterol (mg/dl)	27.61±9.6	32.3±12.29	0.001	28.95±10.63
LDL cholesterol (mg/dl)	76.33±26.9	79.34±26.83	0.391	77.18±26.87
HbA1c (%)	10.12±2.34	10.06±2.55	0.84	10.1±2.4

Data presented as Mean ± S.D,

P-value < 0.05 was considered statistically significant.

The dietary record showed that the protein intake of the subject with diabetic foot ulcer was not appropriate when compared to daily requirement. Mean grams of protein intake was 76.87 in males v/s 56.84 in females. On the other hand protein requirement is much higher than the intake, which is 219.5 (gms) in males and 130.2 (gms) in females as shown in Table-II.

**Table-II:** Protein intake & requirement of the study subjects.

Variables	Male	Female	P-value	Overall
N	364	178	-	542
Protein intake	76.87±26.63	56.84±17.05	<0.0001	70.29±25.68
Protein Required	105.2±28.02	95.43±26.98	<0.0001	101.99±28.04

Data presented as Mean ± S.D,

P-value < 0.05 was considered statistically significant.

**Fig.1:**
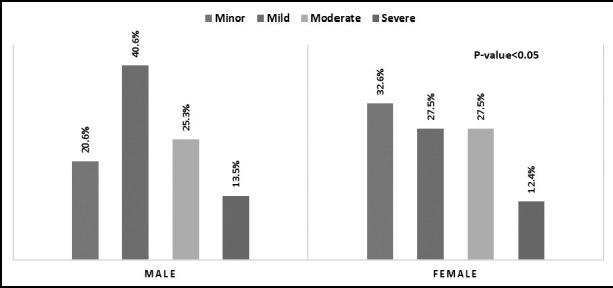
Injury factor category of the study subject.

Almost 66% of the male subject and 55% in female subject were mild to moderate injury factor. The overall significant difference was shown in male to female injury factor category (p<0.05). Fig.1.

**Fig.2:**
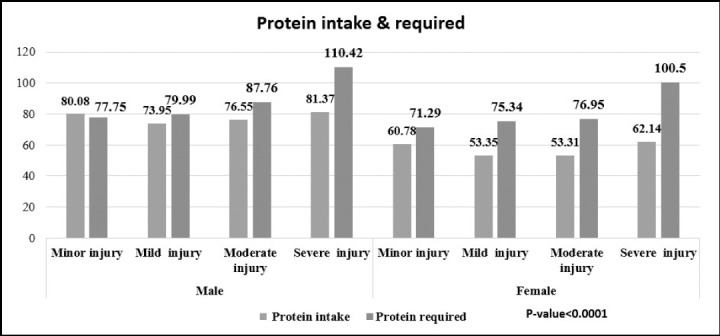
Protein intake and the daily requirements.

The requirement was also calculated according to the injury factor. Only men with minor injury are having increased intake of protein, rest of the patients with higher stages of wound both in men and women were not meeting their requirements as shown in Fig.2 (p < 0.0001).

## DISCUSSION

This study provides information regarding the protein intake and requirement among subjects with type 2 diabetes having foot ulcers. Diet plays an important role in the management of diabetes as hyperglycemia is the leading causes apart from lack of diabetes education that causes non-healing wounds, ulcers and inflammation. Dietary protein is essential for wound healing process.^6^,^16^ Protein is a main source of energy which mainly focuses on the growth and maintenance of the muscles and body tissues.^17^

Results of this study revealed that the dietary intake of the people with diabetic foot ulcer was not adequate as recommended for pressure ulcers. According to National Pressure Ulcer Advisory Panel White Paper, dietary protein is especially important in the elderly adult due to body composition changes that occur with aging and reduced activity levels. The mean age of overall patients included in this study was above 50 years. Therefore, it can be said that apart from the increased protein needs to address the wound, high requirement can be suggested to address age related issues.^15^

Previous studies have reported the importance of dietary protein in the wound healing process but, fewer studies have been conducted in subjects with diabetes having foot ulcer. Majority of studies had determined the protein deficiency in the subjects with wound and foot ulcers. David and Graham study reported about the relationship between wound healing and nutritional state among surgical subjects, observed that the wound healing process was prolonged among malnourished subjects.^18^

One of the study conducted in Maryland between the subjects of pressure ulcer showed that high protein diet improves the healing of pressure ulcers in malnourished nursing home subjects.^7^ Randomized control trial showed the benefit of protein hydrolysate supplement in the residents who have pressure ulcers.^19^ Another study highlighted the importance of protein in wound healing after amputation.^20^ Subjects consuming inappropriate diet will increase time of wound healing so, nutritional states of subjects with diabetic foot ulcer have a significant role in recovery of wound. A study also reported that every third diabetic foot ulcer subjects were malnourished.^21^

Present study observed inadequate intake of protein in both genders as compare to daily protein recommendation. A study conducted in CHINA shows that more than half of the subjects with diabetic foot ulcer were suffering from undernutrition.^22^ In another study researchers observed a mixture of three amino acid arginine, glutamine and β-hydroxy-β-methyl-butyrate had reduced cost of antibiotic and improvement occur in wound healing process.^15^

### Limitations and Strength

There were some limitations in our study. The study was conducted on a small sample size. It was a retrospective observational study and not a prospective, therefore we could not comment on the impact of improved protein intake on the wound. Also the protein intake calculated was on recall basis, which places the question on the actual intake. It was a single center research therefore we could only reach out to a limited number of people and also to the people of similar background. Along with the protein intake micronutrient content could not be checked due to limited budget which could indicate underlying malnutrition and deficiencies. To the best of our knowledge, we couldn’t find any study similar to ours where the patients requirement and actual intake have been observed and calculated. Therefore, despite our limitations our study has some important findings. Further studies are required to validate our findings.

## CONCLUSION

Patients with Type 2 diabetes with foot ulcer do not consume appropriate amount of essential amino acid require for wound healing. There is need of conducting more research to address dietary issues of diabetic foot ulcer subjects. It is important provide information about food choice and determine barriers to follow healthy meal plain. The impact of intervention in those who are protein calorie malnutrition.

### Authors’ Contributions

**NS:** Concept and design, Interpretation of data, prepared and revision of the manuscript.

**ZM, SZ:** Concept and design, editing and revision of manuscript.

**SJ, MA** Interpretation of data prepared and revision of the manuscript.
